# Using OWL reasoning to support the generation of novel gene sets for enrichment analysis

**DOI:** 10.1186/s13326-018-0175-z

**Published:** 2018-02-14

**Authors:** David J. Osumi-Sutherland, Enrico Ponta, Melanie Courtot, Helen Parkinson, Laura Badi

**Affiliations:** 1 European Bioinformatics Institute (EMBL-EBI), Wellcome Trust Genome Campus, Cambridge, CB10 1SD UK; 2 Roche Pharma Research and Early Development, Pharmaceutical Sciences, Roche Innovation Center Basel, F. Hoffmann-La Roche Ltd, Grenzacherstrasse 124, -4070 Basel, CH Switzerland

**Keywords:** OWL, EL, gene ontology, GO, gene set enrichment analysis, enrichment, over-representation analysis, ontology mapping

## Abstract

**Background:**

The Gene Ontology (GO) consists of over 40,000 terms for biological processes, cell components and gene product activities linked into a graph structure by over 90,000 relationships. It has been used to annotate the functions and cellular locations of several million gene products. The graph structure is used by a variety of tools to group annotated genes into sets whose products share function or location. These gene sets are widely used to interpret the results of genomics experiments by assessing which sets are significantly over- or under-represented in results lists. F Hoffmann-La Roche Ltd. has developed a bespoke, manually maintained controlled vocabulary (RCV) for use in over-representation analysis. Many terms in this vocabulary group GO terms in novel ways that cannot easily be derived using the graph structure of the GO. For example, some RCV terms group GO terms by the cell, chemical or tissue type they refer to. Recent improvements in the content and formal structure of the GO make it possible to use logical queries in Web Ontology Language (OWL) to automatically map these cross-cutting classifications to sets of GO terms. We used this approach to automate mapping between RCV and GO, largely replacing the increasingly unsustainable manual mapping process. We then tested the utility of the resulting groupings for over-representation analysis.

**Results:**

We successfully mapped 85% of RCV terms to logical OWL definitions and showed that these could be used to recapitulate and extend manual mappings between RCV terms and the sets of GO terms subsumed by them. We also show that gene sets derived from the resulting GO terms sets can be used to detect the signatures of cell and tissue types in whole genome expression data.

**Conclusions:**

The rich formal structure of the GO makes it possible to use reasoning to dynamically generate novel, biologically relevant groupings of GO terms. GO term groupings generated with this approach can be used in.

over-representation analysis to detect cell and tissue type signatures in whole genome expression data.

**Electronic supplementary material:**

The online version of this article (10.1186/s13326-018-0175-z) contains supplementary material, which is available to authorized users.

## Background

The Gene Ontology (GO) consists of almost 40,000 terms and has been used to annotate millions of gene products to record their subcellular location (e.g., lysosome), their molecular function (e.g., kinase activity) and their wider role in cellular, developmental and physiological processes (e.g., signal transduction) [1]. In its original form, the GO was conceived of as a directed acyclic graph in which terms referring to classes are nodes and edges record relationships between classes including classification (is a) and partonomy (part of). This graph structure is commonly used to group genes annotated with related terms in user-facing tools such as QuickGO [2] and AmiGO [3] and to generate gene sets for enrichment (over- representation) analyses [4] to interpret the results of genomics experiments. For example, an experiment to assess the how treatment of liver cells with a particular drug effects the transcriptome (genome-wide gene expression profile) in liver cells may result in a list of all genes whose expression is increased by the drug treatment. The GO graph structure can be used to group all genes in the relevant genome into sets sharing function or location. One can then ask which gene sets are statistically over or under-represented in the gene list compared to the expected number of of genes from that set in an equivalent length list generated by random sampling from the set of all genes in the genome.

In recent years, the GO has developed into a richly axiomatised formal ontology specified using Web Ontology Language (OWL) [5, 6] and defining GO terms with reference to terms from other ontologies. For example, the GO now records the chemical participants in over 12,000 processes and functions via axioms referencing chemical entities defined by the Chemical Entities of Biological Interest (ChEBI) ontology [7, 8]. Over 8000 GO classes have some direct or indirect logical link to a term from the Cell Ontology (CL) [9] or the Uber anatomy ontology (Uberon) [10]. These record, for example, the location of cellular components (the acrosome and its parts are present only in sperm), cell types that are the sole location of some process (‘natural killer cell degranulation’ only occurs in natural killer cells), and the products of developmental processes (bone is a product of ‘bone morphogen- esis’). There are also over 2500 logical axioms recording the functions of cellular components via links to molecular function and biological process terms.

When combined with standard OWL reasoning technologies, this improved axiomatisation opens up new possibilities for grouping terms and their xannotations in biologically meaningful and semantically precise ways that are potentially useful in enrichment analyses. For example, we can use OWL reasoning queries to group processes occurring in T-cells or in the pancreas, or processes involving nitric oxide or collagen fibers.

The results of enrichment analyses using gene sets for all GO terms can be difficult and slow to interpret due to high levels of overlap between gene sets. There are a number of sources of overlap: grouping via class and part hierarchies means that gene sets derived from annotation to a class subsumes the gene sets of its subclasses and subparts; one GO class can sit in multiple branches of the hierarchy; a single gene product may be annotated to terms in multiple branches. For this reason, many enrichment analyses rely on a more limited number of gene sets, corresponding to grouping under a limited number of high or intermediate level GO terms commonly referred to as a slim.

Rather than use a slim of GO terms, F. Hoffmann-La Roche Ltd. (“Roche”), maintains an internal controlled vocabulary (referred to hereafter as the RCV) for use in enrichment analyses. The RCV consists of around 360 terms, each of which is mapped to a set of terms from the GO. It is tailored to the research interests of Roche, and its terms were chosen with the aim of achieving gene set composition descriptive and broad enough to allow robust and statistically significant results though not so broad and redundant in composition that it prevents easy interpretation of results. Detecting enrichment to gene products involved in anatomy, organ or cell-specific processes or components can be critical for pharmacological research, especially when working with complex tissues where there is a need to tease apart events occurring in specific tissue compartments or cell types. To support this, many RCV terms group GO terms in ways that are out of scope for classes in the GO, including groupings of GO terms related to specific cell, tissue or molecule types.

Here we describe the development and testing of a dynamic, computable mapping between RCV terms and the GO that makes use of OWL reasoning. We show that RCV groupings of GO terms related to specific cell, and molecule types can be used to identify the transcriptomes of those cell types via enrichment analyses.

## Methods

As the RCV is a flat list and includes classifications that are orthogonal to the classification schemes used by the GO, it is not amenable to mapping via ontology alignment techniques that use ontology structure [11]. Given the small size of the RCV, it is viable to manually map each RCV term to an OWL class expression (DL query), which can then be used in conjunction with an OWL reasoner to generate lists of GO terms. The RCV does not include textual definitions to clarify meaning, so for each RCV term we attempted to find a class expression (a mapping query) that reflected the intended meaning of the RCV term, as judged by the RCV term name, manual mappings and discussion with RCV developers.

### Query strategy

We manually mapped each RCV term to an OWL class expression (a mapping query) and used a standard OWL reasoner to generate a combined list of classes equivalent to and classes subsumed by the class expression.

We tested classification and query answering using the GO with imports of CHEBI, CL and Uberon on a 2.9 GHz Intel Core i7 Mac laptop, assigning 10Gb of RAM to the JVM. Classification with the OWL 2 EL reasoner ELK [12] com- pleted in under 6 s and used less than 4Gb of RAM. Subsequent queries of the classified ontology took 10-100 ms milliseconds. In contrast, classification using the HermiT reasoner [13], which supports OWL 2 DL took ~70 min and used 7.5Gb of RAM. Subsequent query answering was very slow, with some test queries timing out. To ensure speed and scalability, we therefore chose to restrict mapping queries to the EL profile of OWL 2 and use the ELK reasoner. The expressiveness of the GO and of imported ontologies is almost entirely within the OWL 2 EL profile (the only exception is a handful of inverse property assertions), so while some incompleteness in query answering is possible, we don’t expect it to be common.

In order to keep the mapping process simple, only a single GO, CL, Uberon or ChEBI mapping class was specified for each mapping query.

To compensate partially for the lack of disjunction (OR) in OWL-EL, we developed a hierarchy of high level object properties for use in queries. For example, we define occurs in OR has participant as a grouping relation allowing queries for processes that occur in a specified cell, or have that cell as a participant. Many RCV terms group processes in which a specified chemical or cell participates with processes regulating those in which it participates (see Table 1 for example). To support such groupings, we used an OWL property chain axiom [5] to define a relation, regulates o has participant, which can be used to query for processes that regulate a process in which some specified entity is a participant. We then defined a super-property, participant OR reg participant, for this new relation and has participant:

**Table 1:** Results table for RCV cannabinoid

GO name	GO ID	manual	auto	checked	black listed	is obsolete
regulation of endocannabinoid signaling pathway	GO 2000124	1	1	1	0	0
cannabinoid signaling pathway	GO 0038171	1	1	1	0	0
endocannabinoid signaling pathway	GO 0071926	1	0	1	0	0
cannabinoid receptor activity	GO 0004949	0	1	1	0	0
cannabinoid biosynthetic process	GO 1901696	0	1	1	0	0

The table shows the mapping of an RCV term“cannabinoid” to a set of GO terms, comparing manual mapping (manual column) with automated mapping (auto column). The automated mapping results from an OWL query for processes in which a cannabinoid participates, or that regulates a process in which a cannabinoid participates. The automated mapping found three additional GO terms compared to the manual mapping. In this case, no manually mapped terms were obsolete in GO and all automated mappings were approved

**regulates** *o* **has participant**

*. subPropertyOf* **participant OR reg participant**

*. . subPropertyOf* **regulates o has participant**

*. . subPropertyOf* **has participant**

These new, high-level object properties are difficult to name in a way that communicates the meanings of mapping queries clearly. In order to compensate for this, we used scripting to generate human readable descriptions for each mapping query. Compare, for example, the mapping query for the RCV term cannabinoid with its description:

***Mapping query***: **participant OR reg participant** *some* cannabinoid.

***Description***: “A process in which a cannabinoid participates, or that regulates a process in which a cannabinoid participates.”

### Ontologies used

We used a fully expressive release version of the GO [14], release version 2015–01-30, supplemented with the bespoke relations described above (21 relations). This resulting ontology includes over 40,000 GO classes and over 13,000 imported classes from the Cell Ontology, ChEBI, Uberon, the Sequence Ontology the Protein ontology and over 130 object properties imported from the OBO Relations Ontology [15]. The DL expressiveness is SRI. For a summary of owl entity and axioms counts please see Table 2.

**Table 2:** Ontology metrics: Counts of OWL entity and axioms types in the ontology used for mapping

entity/axiom type	Count
Logical axioms	142,894
Classes	53,799
Object properties	153
SubClassOf axioms	113,104
EquivalentClasses	29,386
DisjointClasses	148
GCI	6910
SubObjectPropertyOf	164
InverseObjectProperties	28
TransitiveObjectProperty	16
ReflexiveObjectProperty	1
SubPropertyChainOf	46

### Pipeline

Mapping queries were run using the ELK OWL 2 reasoner [12] via calls to the OWL- API [16]. The query and results processing pipeline was written in Jython [17]. All code, mapping tables and results were maintained in a GitHub repository [18]. The mapping was specified using a single tab separated values (TSV) file in which each line maps an RCV term to an OWL-EL mapping query that includes a term from GO, ChEBI, CL, Uberon or NCBI taxonomy [19]. Query results were used to generate a TSV file, allowing direct comparison of manual and automated mappings (see Table 1 for an example). We used the GitHub API to generate tickets for each mapping, linked to the relevant TSV results file, which GitHub renders as a table. This allowed easy manual review and editing by RCV curators at Roche who used the linked tickets to discuss mapping issues and record the approval status of all mappings.

Mapping queries were selected, tested and the results reviewed against manual mappings to decide which patterns were most appropriate. Once a mapping query was chosen, corrections and/or additions to the GO were made where results were wrong or incomplete. At this point, any clear errors in the manual mapping we blacklisted. Review of automated mappings was then passed to Roche who approved or blacklisted individual classes (see Table 1 for an example). When satisfied with the results, the corresponding GitHub ticket was closed, thereby indicating the mapping as approved. Results approved by Roche were combined to produce a new RCV mapping table (available from [20]).

### Over-representation analysis

For each RCV term we generated a gene set consisting of all human genes directly annotated to each mapped GO term (retrieved from NCBI/entrez [21]). These are referred to in the following text and figures using the RCV term name name suffixed with ‘rcv’.

We additionally generated 155 gene sets that are enriched in specific tissue types. These were generated from three datasets: the Neurocrine Biosciences (NB) CNS dataset [22], the GNF Gene Expression Atlas [23] (both based on the *Affymetrix* microarray technology), and sequencing-based RNASeq Atlas [24]). The Gini index was used to identify tissue-enriched genes in each dataset [25] by selecting genes with Gini coefficient *>* 0.7 for the specified tissue. The list of tissue specific genes from each dataset was combined in a non-redundant tissue signature list. These are referred to in the following text and figures by the tissue name suffixed with ‘ts’.

Using tissue type expression data from the Genotype-Tissue Expression project (GTEx) [26], we calculated the average level of expression (mean reads per kilobase of transcript per million mapped reads (RPKM) signal) across all samples available for a given tissue type and used this to construct a gene vector (a rank order list of genes by expression level) for each tissue type. We used the same approach to process a publicly available set of immune-cell type expression data [27, 28] for enrichment analysis.

For each geneset and each tissue-type or cell-type gene vector we calculated an enrichment score, defined as the -log_10_ (*p*-Value) of the Wilcoxon test [29] applied using the entire geneset collection as universe. We present enrichment by using the resulting Z-scores to generate heat maps showing over- or under-representation of each gene set in each tissue, using euclidean distance clustering to cluster similar results on both the X axis (GTEx tissue type) and Y axis (gene set). Heat maps are a standard way to represent this type of analysis, in part because they make clustering of similar results easily visible as blocks of similar patterns.

## Results

### Mapping results

We developed successful mapping queries (owl class expressions) for 308 out of 364 RCV terms and used OWL reasoning to find all classes equivalent to or subsumed by the mapping query for each RCV term (only 72 RCV classes had equivalent classes as well as subsumed classes in GO).

**Fig. 1:**
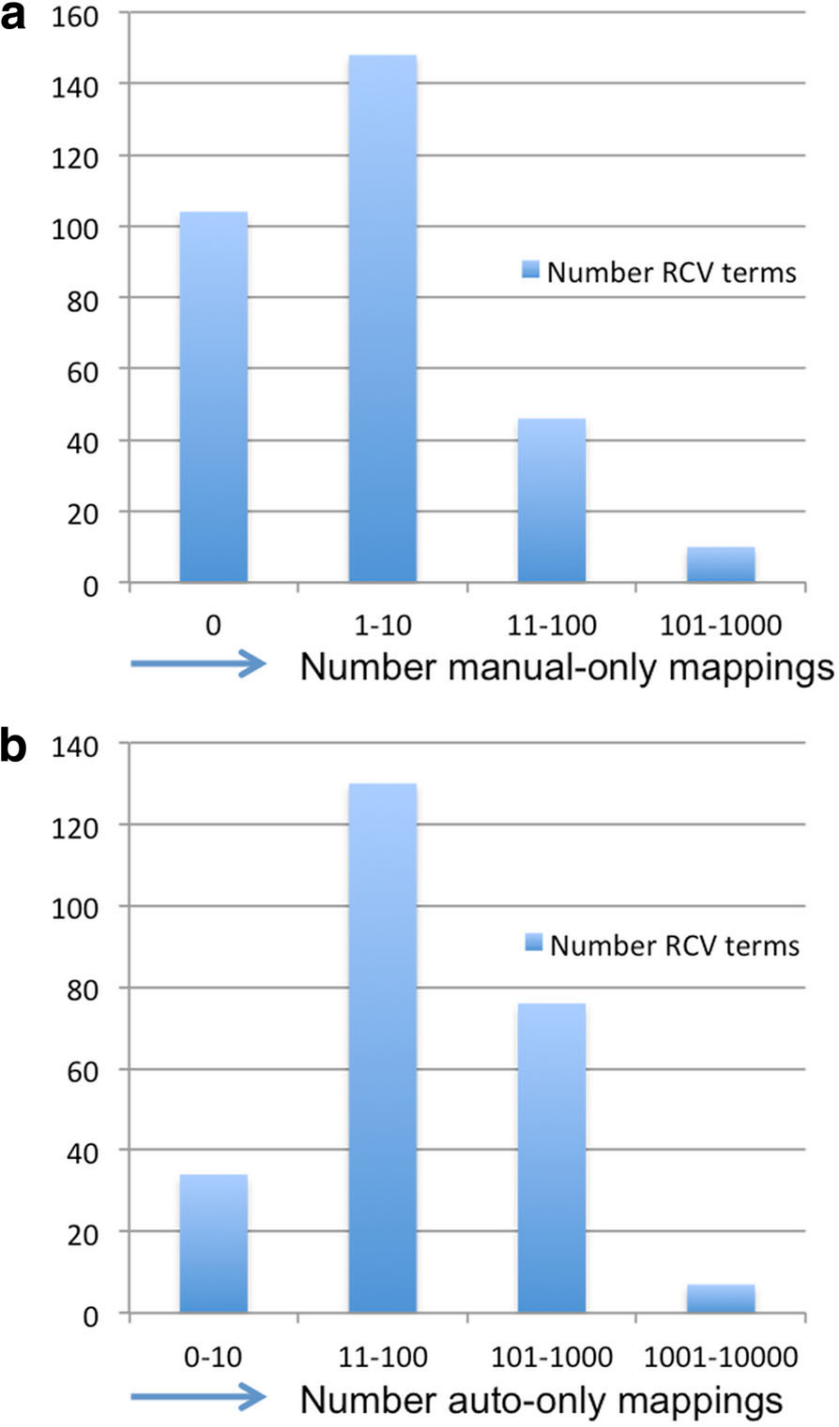
Summary of mapping results **a**. Distribution of manual mappings not found by automated mapping X axis = number of manual-only mappings. Y axis = Number of RCV terms. Over 80% of mappings are completely automated or require less than 10 manual mappings. **b**. Distribution of automated mappings not present in the original manual mapping. X axis = number of auto-only mappings. Y axis = Number of RCV terms. Many new mappings were uncovered by automation

Over a third (104) of the mapping queries were sufficient to recapitulate all manual mappings. A further 40% of the mappings (148) had 10 or fewer additional manual mappings (Fig. 1a) and most of these (114) had fewer than 5.

Mapping queries identified many GO terms that were not in the manual mapping (Fig. 1b). In some cases (e.g., leukocyte activation), over 1000 new mappings were found. On manual review, only 8 out of several thousand automated mappings were flagged as unsuitable by Roche, 56 terms were not mapped. Some were judged to be semantically equivalent to other RCV terms. The rest were rejected as currently not mappable due to the lack of suitable terms or axiomatisation within the GO. For example, RCV has terms for aerobic and anaerobic metabolic processes, but the GO currently has no terms for these processes, and no axiomatization that allows them to be queried for. Further axiomatization of the GO is likely to improve the number of RCV terms that can be mapped.

### Improvements to the GO

While the GO has extensive axiomatization linking processes to cells, anatomical structures and chemicals, this is not always complete. In mapping from the RCV to the GO we found and corrected over 200 omissions in the axiomatization including links from processes to participant cell types, anatomical structures and chemicals. Examples include linking GO process terms referring to the aggregation of immune cell types such as lymphocytes and thymocytes to the relevant cell type terms in the cell ontology.

We also found and corrected a number of errors, including errors in axiomatization of developmental processes that led to incorrect inferences for RCV anatomy terms. For example, we uncovered and fixed errors in axiomatisation of epidermis development that tangled together classification of terms referring to animal epi- dermal structures (e.g. skin and its parts) with those from plants (such as stomatal guard cells).

Assessing the utility of RCV for over-representation analysis.

We assessed the ability of gene sets derived from RCV terms to identify tissue and cell types in enrichment studies. The RCV contains a number of terms for immune cell types defined using a standard pattern that groups terms related to immune cells ina variety of ways. e.g.:

#### T_cells

Some part of a T cell, or some process in which: a T cell participates or that occur in a T cell or which results in the developmental progression of a cell that will form a T cell.

**Fig. 2:**
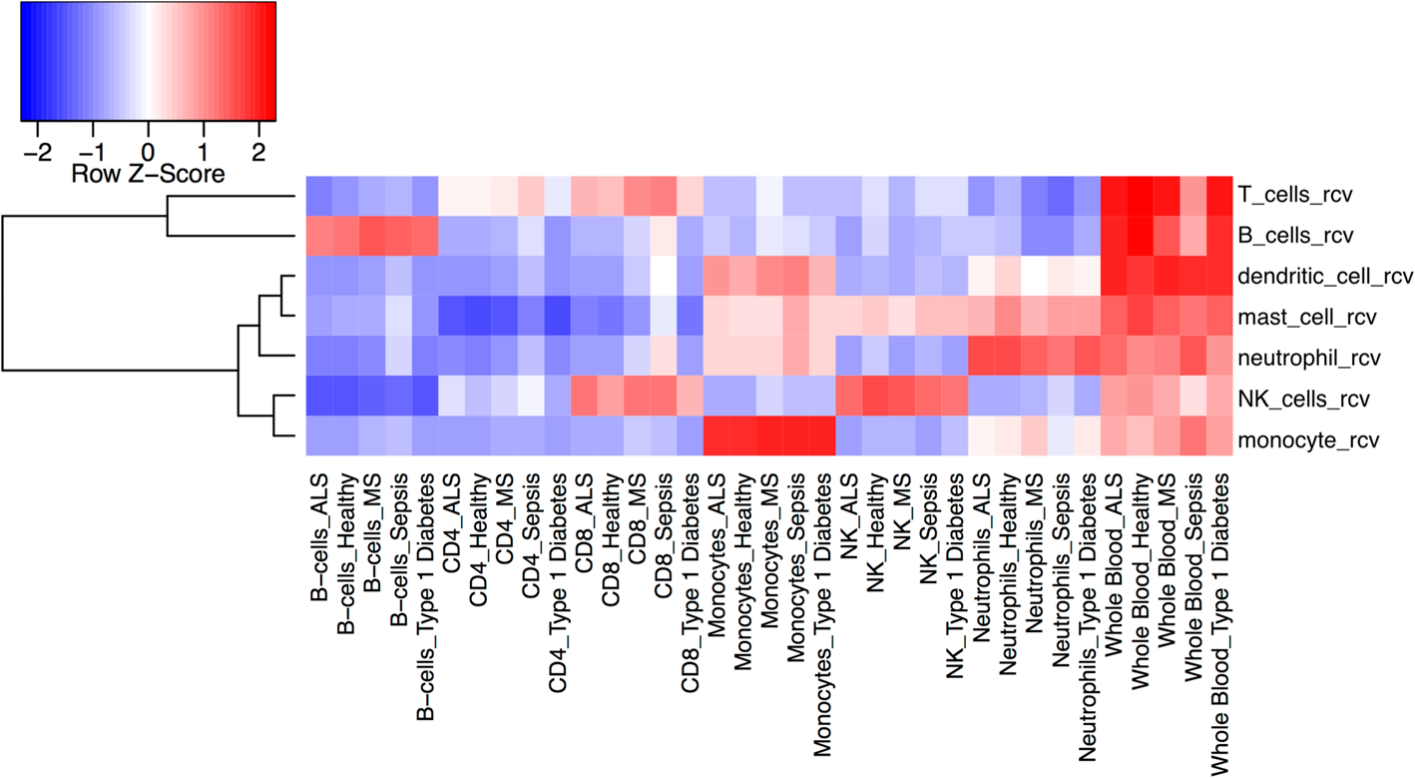
Use of RCV-derived gene sets to identify immune cell types. RCV-derived gene sets (Y-axis); immune cell type transcriptomes (X-axis); over-representation is indicated in red; under-representation in blue. Cell-type transcriptomes are clustered based on similarity of enrichment profile across gene sets

**Fig. 3:**
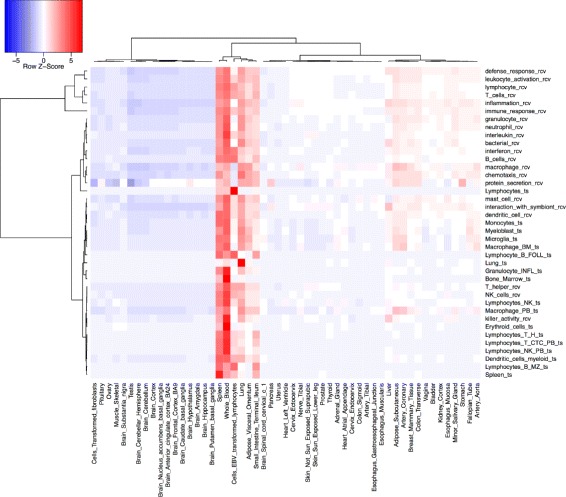
Comparison of RCV derived gene sets and tissue derived gene sets for identification of immune-cell rich tissues Over-representation of RCV-derived gene sets (Y-axis) in tissue-type transcriptomes (X-axis) is indicated in red, under-representation in blue. Tissue-type transcriptomes are clustered based on similarity of enrichment profile across gene sets (X-axis) and gene sets are clustered by similarity of enrichment profile across tissues (Y-axis). Only the immune-rich tissue cluster of gene sets is shown in this figure. For the fully enrichment analysis please see Additional file 1

**Fig. 4:**
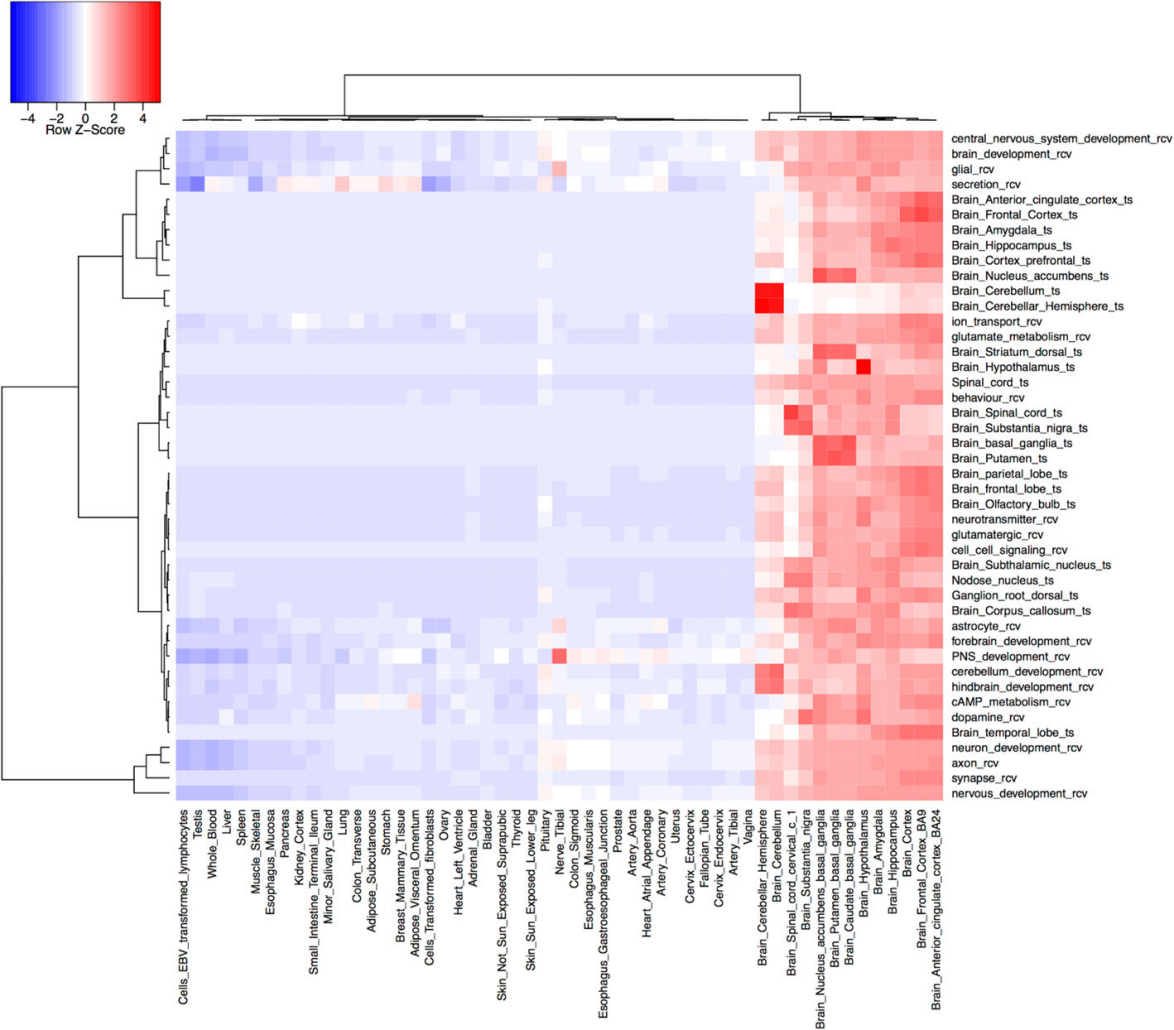
Comparison of RCV derived gene sets and tissue derived gene sets for identification of brain derived tissues. Over-representation of RCV-derived gene sets (Y-axis) in tissue-type transcriptomes (X-axis) is indicated in red, under-representation in blue. Tissue-type transcriptomes are clustered based on similarity of enrichment profile across gene sets (X-axis) and gene sets are clustered by similarity of enrichment profile across tissues (Y-axis). Only the brain tissue cluster gene sets is shown in this figure. For the full enrichment analysis please see Additional file 1

Using immune cell-type expression profiles from [27, 28] we calculated how over-or under-represented each RCV immune cell type gene set was in each cell-type expression profile. The results are displayed in Fig. 2, with cell-types (X-axis) clustered according to similarity of enrichment profile across gene sets. All RCV immune cell gene sets are highly enriched in whole blood transcriptomes. The over- representation profiles for RCV immune cell-type gene sets matched immune cell types. NK cells rcv and B cells rcv and monocytes rcv were all enriched only in matching cell-type transcriptomes. CD8 is expressed in T cells and a subset of Natural Killer cells [30, 31]. Consistent with this, enrichment to the T cells rcv and NK cells rcv gene sets is seen in CD8 expressing cells. CD4 is expressed in subset of T-cells [30]. Consistent with this, a low level of enrichment is seen for the RCV T-cell gene sets. To test the ability of the RCV to match tissue types more broadly, we used tissue-type expression profiles from GTEx [26], a publicly available data set with expression profiles of 47 different tissues. We used this to compare enrichment to RCV terms to enrichment to a set of tissue-derived gene sets. The complete results are available in Additional file 1 as a heat map showing over and under representation of each gene set (Y-axis) for each GTEx tissue-type (X-axis), with both axes clustered for similarity. Distinct enrichment clusters generated by this analysis include clusters identifying tissues rich in immune cells (Fig. 3), clusters indentifying brain tissue (Fig. 4 and clusters identifying skin (Additional file 1).

Figure 3 shows enrichment analysis for genesets related to immune cells. RCV immune cell genesets form a co-cluster with immune cell enriched genesets - showing over-representation in tissues rich in immune cells such as blood, lungs and gut mucosa as well as in transformed lymphocytes.

Comparable ts and rcv gene sets have very little overlap: all comparisons between equivalent rcv and ts genesets have a Jaccard index of less than 0.1 (Table 3). They therefore provide complementary sets of signatures for detecting the presence of immune cells in samples for which transcriptomic data is available.

**Table 3:** Overlap between cell-specific gene sets derived from RCV and cell expression data is low

Gene sets	Jaccard Index
B cells rcv vs Lymphocyte B FOLL ts	0.064
NK cells rcv vs Lymphocytes NK ts	0.000
T cells rcv vs Lymphocytes T various ts^a^	0.025^a^
T helper rcv vs Lymphocytes T H ts	0.032
dendritic cell rcv vs Dendritic cells ts	0.000
granulocyte rcv vs Granulocyte INFL ts	0.082
lymphocyte rcv vs Lymphocytes NK ts	0.071
macrophage rcv vs Macrophage PB ts	0.033
mast cell rcv vs Mast cell PB ts	0.045

Column one lists the two gene sets compared. Column 2 lists the Jaccard similarity coefficient comparing the two gene sets (0 = no overlap, 1 = full overlap.) ^a^In the case of T cells the average of a range of T-cell expression datasets is shown

Also included in the cluster are immune-system related groupings such as immune response rcv, inflammation rcv and leukocyte activation rcv, showing that the RCV provides a semantically richer picture than simply detecting cell-types.

Figure 4 shows a similar cluster of enrichment for brain tissue samples. Gene sets derived from annotation to processes involving glial cells (glial rcv) and more specifically astrocytes (astrocyte rcv) are sufficient to distinguish brain tissue types and nerves from other tissue types in GTEx. The cluster also contains RCV gene sets for novel grouping terms defined with respect to molecules (dopamine, cAMP, neurotransmitter) and cell components (synapse) with definitions following the patterns:

#### Neurotransmitter

A process in which some substance with neurotransmitter biological role participates, or that regulates a process in which substance with neurotransmitter biological role participates.

#### Synapse

A synapse OR part of a synapse OR a process that results in organisation of a synapse OR that has a synapse as a participant.

These enrichments make sense given what is known about the biology of neural tissue, as do a set of RCV terms that map to conventional GO terms: ion transport; cell cell signaling, glutamate metabolism and nervous system development terms.

## Discussion

Mapping of RCV terms to set of GO terms is now fully dynamic, allowing RCV to be automatically kept up to date as the GO. Where new terms follow mapping query patterns that are already used, they can be added simply by specifying an additional line in the mapping file.

48% of mapped RCV terms have 10 or fewer manual mappings. We are reviewing all of these cases to decide whether to drop manual mappings or whether complete automation might be achieved by a different query strategy. In some cases, a more complete mapping could be achieved by a disjunctive query. For example, all RCV terms referring to metabolism of some specified chemical are mapped to GO terms referring to metabolic and transport processes in which the specified chemical is a participant. (This is consistent with some medical use of the term metabolism.) A more complete mapping could be achieved using a disjunctive query with an OWL2 DL reasoner such as HermiT. This approach was found to be prohibitively slow but new generation reasoners such as MoRE [32] which combine ELK with DL reasoners such as HermiT may turn out to be useful in this approach. A simple, if potentially incomplete alternative would be to simply run two EL mappings using ELK and generate a union of the results.

56 terms were not mapped. Some were rejected from the pipeline as they were judged to be too close in meaning to other RCV terms. The rest were rejected as currently un-mappable due to the lack of suitable terms or axiomatisation within the GO at this time. For example, GO currently has no formal way to group aerobic or anaerobic metabolic processes, although it does reflect the aerobic or anaerobic nature of many metabolic processes in their names and textual definitions.

### Making novel groupings of GO terms generally accessible

The approach described here could be used to provide a view of the GO that groups terms in ways defined with reference to the complete range of cell-types, chemical types and anatomical structures referenced by the GO and all of their ancestor classes. This is already reflected in some of the newer functionalities of the GO browsing tool AMIGO, which now displays inferred annotations to cell-types based on axioms in the GO recording where processes occur [33]. An extended version of the GO with extended axiomatisation and imported terms from external ontologies includin CL, Uberon, ChEBI is available from [34].

The system described bears some relationship to TermGenie [35] which is already used to generate 80% of new GO terms. One possible approach to fulfilling the needs of external groups for types of classification not included in the GO would be to offer a TermGenie-like system to create bespoke terms.

## Conclusions

Our work demonstrates how the logical structure of the GO can be used to achieve biologically meaningful mappings between concepts in external controlled vocabu- laries and corresponding sets of GO terms, even when there is no concept in the GO that is directly equivalent the term to be mapped. This is possible as long as the concept can be mapped to an OWL class expression referencing classes and relations in the full version of the GO. These classes may come from the GO, or from ontologies from which the GO imports classes such as ChEBI, CL and Uberon. The resulting mapping is dynamic and so can easily be kept up to date as the GO evolves.

While OWL 2 DL profile queries could be used for these mappings, this would make mapping software slow to run, resource intensive, and may not be sustainable as the GO becomes still larger and more complex [6]. The mapping system we describe uses class expressions restricted to the OWL 2 EL profile to ensure that mapping is fast and scaleable. It also demonstrates how OWL property chains and property hierarchy can be used to partially overcome the absence of disjunction (OR) in OWL 2 EL.

The RCV includes many terms that group GO terms in novel ways by their rela- tionship to some type of cell, molecule, tissue or cell component. One possible usage of these terms is to provide a mechanism for detecting the signatures of particular cell or tissue types in transcriptomic data. We demonstrate the effectiveness of this by showing how gene sets derived from RCV terms for cell types can be used to identify specific immune cell types and how gene sets derived from RCV terms for cell, molecular and cell component types can be used to identify tissue types. In some cases, gene sets derived directly from transcriptomic data may be used for the same purpose (see Figs 3 and 4). In these cases the RCV term sets provide an alternative method using gene sets with very little overlap to those derived from differential expression (Fig. 3). Unlike gene sets derived from transcriptomic data, those derived from the GO and its annotations are not limited by the ability to experimentally isolate suitable biological samples and can include broad groupings of cell or tissue types that are unlikely to be isolated together (e.g. all glial cells (Fig. 4), or all epithelia).

## Additional file

Additional file 1: A complete over-representation analysis for RCV gene sets against GTEx tissue type transcriptomes. The analysis is displayed as a heat map with RCV on the Y-axis, GTEx on the X-axis, over-respresentation in blue and under-respresentation in red. Both axes are clustered for similarity (see Methods for details). (PDF 91 kb)
