# Supplementary figures and images for: Using OWL reasoning to support the generation of novel gene sets for enrichment analysis
Source: J Biomed Semantics. 2018 Feb 14;9:10. doi: 10.1186/s13326-018-0175-z (PMC5813370; doi:10.1186/s13326-018-0175-z)

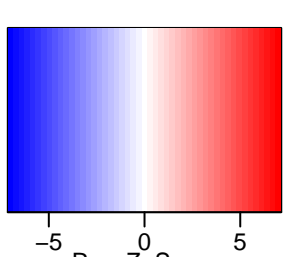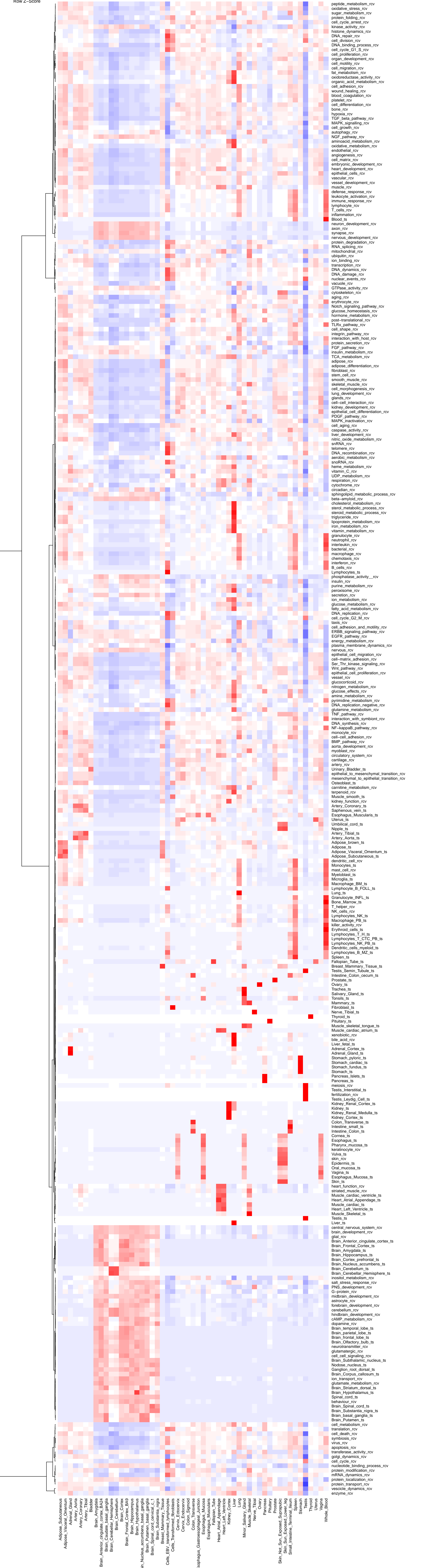

Supplement: Supplementary file 1 — A complete over-representation analysis for RCV gene sets against GTEx tissue type transcriptomes. The analysis is displayed as a heat map with RCV on the Y-axis, GTEx on the X-axis, over-respresentation in blue and under-respresentation in red. Both axes are clustered for similarity (see Methods for details). (PDF 91 kb) [file 13326_2018_175_MOESM1_ESM.pdf]
